# Applying Factorial Modeling to the Optimization of Textural Descriptors and Sensations Relative to Obtaining the Experimental Prototype of Chickpea Puree with Avocado

**DOI:** 10.3390/foods14234082

**Published:** 2025-11-28

**Authors:** Maria Lidia Iancu, Ion Dan Mironescu

**Affiliations:** Department of Agricultural Sciences and Food Engineering, The “Lucian Blaga” University of Sibiu, Faculty of Agricultural Sciences, Food Industry and Environmental Protection, 5-7, Ion Raţiu Street, 550024 Sibiu, Romania; ion.mironescu@ulbsibiu.ro

**Keywords:** puree, avocado, chickpea, factorial model, textural analysis, quality indicators

## Abstract

The aim of this study was to obtain an optimized recipe for a chickpea avocado puree food prototype. The optimization was performed using a model constructed from the results of a 2^3^ factorial experimental design with central points. The model correlates three factors set through the recipe—chickpea content, avocado content and sugar-to-vinegar ratio- with some measurable quality indicators—instrumentally determined textural descriptors, sensorial determined textural descriptors and acidity. The nine recipes for the chickpea and avocado puree corresponding to the 2^3^ experiments and the central point were compared in order to select the best candidate for the prototype. The statistical analysis of the results through ANOVA has shown that the instrumental and sensorial texture indicators are influenced by ale factors and al interactions between them (all combination of two and all three) (*p* < 0.05). The influence of vinegar-to-sugar ratio and of the interaction of all three factors on acidity in the current experimental context cannot be statistically sustained (*p* > 0.05). The significance of the coefficients of the regression model was also statistically confirmed. The destabilizing force had values between 59.5 g and 108.3 g, the total acidity (expressed in acetic acid grams/100 g product) was between 0.8 and 1.62 and the sensory scores were between 8.8 and 11 points out of a maximum of 25 points. The recipe that was chosen as optimal, both for its sensorial and chemo-physical properties, (instrumental texture and acidity) was that corresponding to experiment 1. The recipe is based on an equal proportion of chickpeas (x_1_) and avocado (x_2_) and with a ratio between sugars and acid (x_3_) of 32.5. The values of the force (Y_1_) for exp.1 was 63.7 g and, according to the mathematical model, the most significant influence is of the chickpea and avocado quantities and sugars/acids ratio. The cohesion forces between the ground chickpea particles and avocado resulting from the protein–lipid interaction influenced the textural properties (hardness, spreadability, consistency and creaminess) to a cumulative value of 2.75 out of a maximum of 5. The product obtained following the recipe corresponding to experiment 1 has an acidity (expressed as gram of acetic acid/100 g product) of 0.960 and a humidity of 74.9% (the highest of all recipes). The product has the user acceptability and the quality indicators needed for mass production and commercialization. It can contribute to the increase in avocado consumption and popularity in Romania.

## 1. Introduction

Puree is defined as a viscous, soft, spiced, sweet and sour mixture obtained from ripe fruits that is consumed in a combination with other foods of animal origin or just with bread [1]. It is widely accepted by consumers and contributes to economic growth, reducing losses and commercial depreciation of harvest that can no longer be consumed fresh [2]. The most popular puree is that made from tomatoes [1]. With the aging of the global population, the number of people who need finely textured mashed foods (purees) with high nutritional value has increased [3]. The combination of avocado (fruit) and chickpea (legume) can be chosen due to nutritional and economic reasons.

Avocado (*Persea americana*) is widely popular and consumed throughout the world. There are farms that grow subtropical or tropical fruits rich in omega-6, such as avocado [4]. The largest producer is Mexico [5]. Consumption is also facilitated by the climate conditions that have influenced the behavioral theory of the consumers [6]. Thus, avocado is consumed in combination with tomatoes, as ketchup [7] and as salad dressing [8]. The avocado fruit is climacteric, perishable and in India it is called “butter fruit” [9]. It has a creamy, buttery texture that works as a lubricant [10]. It has high nutritional value, contains fatty acids, hydroxycinnamic acids, hydroxybenzoic acids, flavonoids, proanthocyanidins, procyanidins, phenolic alcohol derivatives and carotenoids with good antioxidant activity [11,12]. Avocado in the form of puree can be a good partial substitute for wheat flour in cookie recipes, as a fat substitute. It influences the texture, sensory characteristics and chemical composition of food products. It has also been noted to influence the hydration capacity of colloidal mixtures and the moisture content of muffins [13,14]. Avocado, ready to eat, has textural properties that are influenced by moisture and storage time. Avocado pulp is pasty and creamy and the gumminess influences the crushing properties and the destabilizing force required to create swallowing properties. The very important textural parameter for avocado pulp is firmness = 3.29 g [15]. In various food systems, avocado, through its varieties, can be used as a functional ingredient [16].

Chickpea (*Cicer arietinum* L.) is an essential legume, whose dried grains are consumed [17]. It is the second-most important family of crop plants from an economical point of view, after the cereal crops [18]. India is the market leader followed by Pakistan and Turkey [19]. Chickpea is rich in nutrients, especially proteins and fibers, vitamins, minerals and complex carbohydrates. During the heat treatment, there are losses of nutrients. Thus, the moisture remains the same; 10% of the lipid content decreases to 4.33% (flour) or 1.47% (chickpea puree), the protein content decreases from 18% to 7.13%, whereas the dietary fiber content drops from 17.86% to 6.24%, the ash from 2.99% to 1.03% and the carbohydrates from 49.15% to 14.3% [20]. The rheological and sensory properties of chickpeas can be altered during the technological operations such as crumbling or the heat treatment [21]. Chickpeas help improve the intestinal integrity; it has an anticancer, antioxidant and anti-inflammatory activity. They are consumed after the grains are subjected to hydrothermal and mechanical operations. The most popular ways of consuming chickpeas are as follows: hummus, beverage, snacks, pancake, pasta, noodle, or cooked seeds [22]. Chickpea puree is also used as a fat substitute in food materials along with hydrocolloids [23] and gluten-free products [24]. As shown through textural analysis performed using TA.XTPlus Texture Analyzer, the addition of chickpea puree modifies the destabilizing force for mixtures. The force decreases with the increase in the chickpea amount. Chickpea puree also decreases the moisture content of mixtures. Products with added chickpea were positively appreciated by panelists in sensory testing [24].

The long-term preserved, ready-to-eat legume puree, characterized by high service value and long shelf life, could represent another effective solution for increasing consumption.

Advanced grinding operations specific to purees substantially influence food texture [25]. Consistency measuring is a usual textural analysis of these simple mixtures with added spices or thickeners [26]. The analysis of vegetable purees is performed through instrumental textural analysis, sensory and physicochemical analysis [27]. Textural descriptors such as friability, consistency (N.s), adhesiveness (N.s) and cohesiveness (N) are associated with features of the force–time curves resulting from the instrumental methods for texture assessment. Firmness and consistency are determined on the positive area of the force–time graph (F(g)-time(s)) of an instrumental analysis; adhesiveness, cohesiveness and stickiness are in the negative area [27]. The firmness is the positive maximum of the force in the graph recorded by a texturometer [28]. These characteristics can also be assessed through sensory analysis using analytical methods with an unstructured scale [27].

The purees can be customized with a large variety of ingredients. We choose to develop a prototype for a spreadable puree from a mixture of chickpeas and avocado. To select the optimal recipe for the chickpea and avocado puree, we used a mathematical model derived from the results of a factorial experimental design. The model describes the influence of three independent variables (amount of chickpeas, amount of avocado and ratio between the sugar content and acid content) on three dependent variables (strength, total acidity and sensory note). Based on technological considerations the experimental work included instrumental textural analysis, total acidity, moisture measurements and sensory analysis were used to assess the consumer acceptance.

## 2. Materials and Methods

### 2.1. Materials

#### 2.1.1. Raw Materials

For the preparation of the puree, the following materials were used: fresh but very ripe avocado fruit, canned chickpeas (preserved by heat treatment), 9° vinegar, salt, garlic, jars and lids that were purchased from stores.

#### 2.1.2. Preparing Avocado and Chickpea Puree

Freshly ripe and undamaged avocados were peeled, pitted and finely mashed. The mashed avocado was combined with finely chopped boiled chickpeas using the BAMIX (Bamix, Switzerland) Gastro 350 Multifunctional Mixer. The ingredients such as vinegar, sugar, salt and garlic were added and the blender was used again to obtain a very smooth composition. The blending speed was 1800 rpm for 5 min. We started from the following recipe (chickpeas 450 g; 440 g avocado; 15 g sugar; 8 g vinegar; 15 g salt; 14 g garlic) for the preparation of 942 g of puree and varied the composition to obtain the experiments of the factorial design. The corner points of the 23 factorial experiments were coded as exp.1, exp.2, exp.3, exp.4, exp.5, exp.6, exp.7, exp.8. The replicates for the central point were coded as exp.9, 9′, 9′′. In the table with compositional design presents the composition for each of the experiments. The 27 samples (three replicates for each experiment and the three replicates of the central point) were dosed into jars with a capacity of 165 g and pasteurized according to a (5′-10′-5′)/90 °C heat treatment scheme (i.e., 5 min warm-up to 90 °C; 10 min hold at 90 °C; 5 min cooling). The pasteurization was performed using a thermoregulated pasteurizer, Adler AD 4496, 2600 W, with a capacity of 28 L. After cooling, the product was kept at a temperature between 15 and 20 °C. After unsealing, it is recommended to store the product at a low refrigeration temperature.

### 2.2. Methods of Analysis

#### 2.2.1. Methods

For the analysis of the raw materials, avocado and chickpeas, as well as for the obtained purees, the following analysis methods were used: organography [29], moisture content (the thermobalance A&D M5 50) (%), total dry matter (100 m) (%) (m-moisture) [30], titratable acidity (expressed as citric or acetic acid g/100 g) [31], soluble solid content (TSS) (°Brix) [32], pH-value (pH-meter, Orion Tip 2-STAR, England) [33], vitamin C (mg/100 g) [34], salt content (%) [35] and net mass (g) [36], and for the calculation of important technological indicators, the following formulas were used: (l_t_ = $\;\frac{W_{in}-W_{out}}{W_{in}}\cdot 100$, (%); yield production, Y_p_ = $\frac{W_{j}}{W_{O}}\cdot 100$, (%); the specific raw material consumption c_smaterials_ = $\frac{W_{O}}{W_{j}}$, (kg/kg); in which l_t_—total technological losses; W_in_—weight entered; W_out_—output weight; Y_p_—production yield; W_j_—weight puree; W_O_—weight material reception; c_smaterials_—specific consumption of materials.

#### 2.2.2. Textural Analysis

The texture of the samples was measured at ambient temperature of 25 °C using the texture analyzer TA.XT PlusC, (with digital interface and software for Windows 10 operating system). A penetration test simulating oral processing was used to assess the destabilizing force for the avocado–chickpea puree mixture. The samples were placed in plastic containers and the air bubbles were removed. A 60° cone (P/60°) was used. The measured resistance force (g) is correlated with textural descriptors like spreadability, stickiness, hardness and firmness.

#### 2.2.3. Multidimensional, Factorial Study

We selected a factorial experimental which facilitates the deriving of a model with a minimum number of experiments. Because we identified 3 main factors, a 2^3^ design using two levels for each factor seemed adequate for the development of an optimal prototype—a preliminary exploratory phase. To test for curvature and possible nonlinear effects, we also included a central point. The two levels in the factorial experiment are coded as −1 and +1, that is, higher and the central point as 0. For these experimental data, the model that correlates the independent and dependent variables was formulated as follows. The independent variables are as follows: chickpea content—x_1_; avocado content—x_2_; (sugar/vinegar) ratio—x_3_. The dependent variables we chose in this study are as follows: strength—Y_1_; sensory note—Y_2_; total acidity—Y_3_.

The equation of the model for each type of response is as follows:

(1) Y_0_ = b_0_ + b_1_·x_1_ + b_2_·x_2_ + b_3_·x_3_ + b_4_x_1_x_2_ + b_5_x_1_x_3_ + b_6_x_2_x_3_+ b_7_x_1_x_2_x_3_

where b_0_—value of the adjusted response at the central coordinate point (0, 0); b_1_, b_2_, b_3_—linear limit of the regression; b_4_, b_5_, b_6_—2nd order interaction coefficients, b_7_—3rd order interaction coefficients.

The experimental results were further investigated through ANOVA (analysis of variance) to assess the influence of the factors and of their interactions on the dependent variables. ANOVA was performed for the 24 samples (3 replicates for each of the 8 experiments of the factorial design). ANOVA was performed using the R open-source software 2020. The experimental data sets corresponding to each of the three models were structured in data frames. Then ANOVA models were fitted on each of these data frames. A summary of these models returned the *p*-values corresponding to each factor and factor interaction of 2nd- and 3rd-order. A *p*-value lower than 0.05 confirmed that the corresponding factor or factor interaction has influence on the dependent variable.

Regression was used to fit a statistical model on the experimental data.

The calculation of regression coefficients is performed using the following formulas:

(2) $$b_{i}\;=\;\frac{\;\sum_{i=1}^{N\;}x_{ij}y_{i}}{N}$$

where b_j_—regression coefficient; x_ij_—independent variable; Y_i_—dependent variable or response variable; N—number of experiments of the model.

Calculation of Y_medium_ is performed at point “0”. The 3 observed and measured replicates are used to test for homogeneity. The “*t*-student” test is used, the standard deviation (s_e_) of the values of the dependent variable studied and the standard deviation (s_b_) are calculated at point “0” using the following formulas:

(3) $$s_{e}^{2}=\;\frac{\sum_{i=1}^{3}{\left(Y_{i}^{o}-Y_{mediu}^{o}\right)}^{2}}{3-1}$$

(4) $$s_{b}=\frac{s_{e}}{\sqrt{N}}$$

where $Y_{mediu}^{o}$ is the average of the replicates observed at the point “0” or the dependent characteristic; $Y_{i}^{o}$—the dependent characteristic being studied.

(5) $$t_{j}=\frac{{|b}_{j}|}{s_{b}},$$

where t_j_ is the significance of the coefficients can be tested using Student’s *t*-test.

The regression equation is estimated to see how well it fits the observation using Fisher’s test, that is, the variance ratio.

The square of the means of the remaining values (residuals) is as follows:

(6) $$s_{r}^{2}=\frac{\sum_{i=1}^{8}{\left(Y_{i}-Y_{icalc}\right)}^{2}}{N-l}$$

The significance of the coefficients is tested using Student’s “t” test. Where s^2^_r_ the mean square of the residuals,

(7) $$F_{1}=\frac{s_{r}^{2}}{s_{e}^{2}}$$

where F_1_ is the ratio between the square of the remaining values and the square of the means at the point “0”.

The Fischer test was used to demonstrate that the chosen model is good [37,38].

#### 2.2.4. Sensory Analysis

For sensory analysis, the method of comparison with unitary scoring scales is used [39]. The organoleptic characteristics evaluated are appearance, color, taste, smell and consistency. They are compared with scoring scales from 0 to 5. For each characteristic, the following was calculated:

AS_mp_ = AS_m_ × f_i_ × f_t_,

where AS_m_ = AS_mp_ is weighted average score; f_i_ = importance factor, f_t_ = 4, transformation factor AS_mp_ is weighted average score. The total average score (AS_mt_) is then calculated by summing the weighted average score values from all organoleptic characteristics. A questionnaire will be completed by each panelist. A number of 25 panelists, boys and girls aged between 21 and 25 years, students of the Faculty of *Agricultural Sciences, Food Industry and Environmental Protection of the “Lucian Blaga*” at the University of Sibiu, were used. For each characteristic, the scoring steps are defined as in Table 1. The panelists are specifically trained. They received questionnaires (Supplementary Materials) and approximately 20 g of each sample at each tasting. The clear definition of the characteristics of the samples and the intermediate values between 0 and 5 were established following training sessions of 2 h/week for 30 days. During this time, the samples were tasted, the sensations obtained were interpreted and discussions were held with the panel leader. The samples are coded for sensory analysis as follows: 736—experiment1 (exp.1); 763—exp.2; 637—exp.3; 183—exp.4; 187—exp.5; 873—exp.6; 137—exp.7; 667—exp.8; 831—exp.9, 9′, 9′′.

## 3. Results

The developed model correlates the composition of the puree variants corresponding to the factorial design with practically determined, experimental values of some output quantities that characterize the quality of the purees.

### 3.1. Technological Studies

#### 3.1.1. Technological Flow in the Manufacture of Chickpea Puree with Avocado

**Figure 1:**
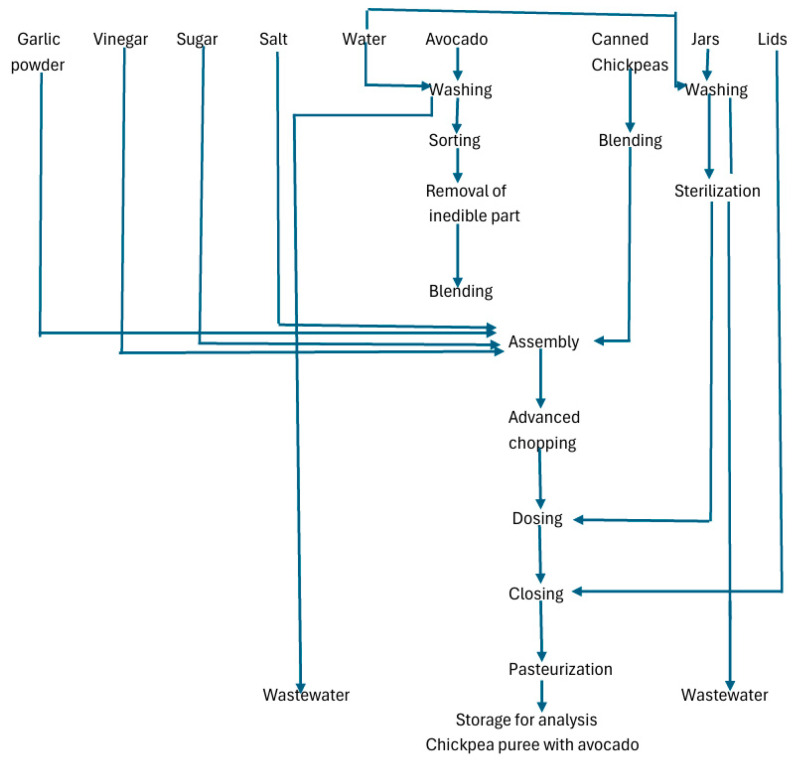
Technological flow for obtaining chickpea puree with avocado and materials.

Figure 1 shows the stages of conditioning the raw materials and producing the chickpea puree with avocado for the variants proposed by the factorial design The basic technological indicators for obtaining chickpea puree with avocado are the production yield calculated according to the formulas in point 2.3, which is 75 ± 3.135% and total losses, 21.2 ± 2.97%.

#### 3.1.2. Physicochemical Indicators for Canned Chickpeas and Fresh Avocado

The results of the physicochemical analyses for canned chickpeas and fresh avocado fruit are shown in Table 2 and were carried out following the instructions of the methods presented in Section 2.2.1. These are real and significantly variable. The value of *p* is ˂0.00001. The result is significant at *p* ˂ 0.05.

The characteristics of the avocado fruit are those that comply with the standard and fall into the quality class for consumption. In other studies, the following values were obtained: for moisture = 68.9–75%, total dry matter = 25–31.5%; and for chopped avocado pulp, pH = 4.16, total acidity 0.19 mg/mL [40]. The values are influenced by the degree of ripeness, variety and storage conditions, factors that were not studied here. Canned chickpeas have characteristics corresponding to those of products obtained and preserved by heat treatment. The two fractions (liquid and solid) are used for the preparation of the puree.

#### 3.1.3. Experimental Model for Creating a Prototype of a Chickpea and Avocado Puree Product Using Factorial Analysis

The development of a mathematical experimental model for improving the ratio of the component materials was performed through statistical optimization. In order to streamline the experimental design, we used a dimensionless coordinate system which uses “+1” for the level above and “−1” for the level below the central point coded by “0”. The system is presented in Table 3.

The ingredients and stages from the technological scheme presented in the technological studies subchapter were used for preparation of the experimental samples. The combination of factor (independent variables x_1_, x_2_, x_3_) values which define each experiment of the factorial design are presented in Table 4. The other ingredients in the recipe such as salt and garlic are added in constant quantity.

ANOVA performed on the experimental data has shown that on force (Y_1_) and on the sensorial attributes (Y_2_), all factors (x_1_, x_2_, x_3_) and all interactions (x_1_x_2_, x_1_x_3,_ x_2_x_3,_ x_1_x_2_ x_3_) have had influence (*p* < 0.005). The influence of x3 (sugar/vinegar ratio 0) and 3^rd^-order term (x_1_x_2_ x_3_) on acidity are not statistically sustained (*p* > 0.05).

The equation model for each type of response is the one presented in Section 2.2 together with all the formulas for calculating the regression coefficients and performing the homogeneity test at level “0”.

The coefficients for the linear equations of the three models are summarized in Table 6.

**Table 5:** Compositional experimental design and dependent variables’ response.

					Response					
Experiments	x_0_	x_1_	x_2_	x_3_	* Y_1_	Y_1calc_	** Y_2_	Y_2calc_	*** Y_3_	Y_3calc_
1	+1	−1	−1	−1	63.7	66.350	11	10.75	0.960	1.0925
2	+1	+1	−1	−1	80.80	80.8	9.76	9.8863	1.200	1.2339
3	+1	−1	+1	−1	59.500	51.75	8.8	9.1387	1.320	1.1875
4	+1	+1	+1	−1	91.600	91.6	9.7	10.01	0.800	0.6673
5	+1	−1	−1	+1	108.300	108.3	10.50	10.011	0.800	0.9342
6	+1	+1	−1	+1	75.60	75.6	9.3	9.387	1.270	1.4027
7	+1	−1	+1	+1	75.8	75.6	10.19	9.8863	1.020	1.9851
8	+1	+1	+1	+1	80.8	83.8	10.34	10.7589	1.620	0.5877
9	0	0	0	0	81.2		10.46		1.020	
9′	0	0	0	0	80.3		10.00		1.010	
9′′	0	0	0	0	81.1		10.03		0.998	

x_0_—dummy variable x_0_ = +1; * destabilization force; Y_1calc_—the value of the response equation with the interaction of coefficients, for force; ** sensory note; Y_2calc_—the value of the response equation with the interaction of coefficients for the sensory score; *** total acidity, dependent measured quantities; Y_3calc_—the value of the response equation with the interaction of coefficients for the total acidity.

**Table 6:** Value of polynomial regression coefficients for each dependent variable studied and calculated.

Regime Coefficients	Y_1calc_	Y_2calc_	Y_3calc_
b_0_	79.8625	9.9488	+1.1238
b_1_	3.0875	−0.1738	+0.0988
b_2_	−2.2375	−0.1913	+0.0663
b_3_	5.9625	0.1338	+0.0538
b_4_	6.9875	0.4363	−0.0788
b_5_	−9.2125	−0.0887	+ 0.1688
b_6_	−3.8875	0.3738	+ 0.0763
b_7_	3.2375	−0.0987	+ 0.1113

Y_1calculated_—force; Y_2calculated_—sensory score; Y_3calculated_—total acidity.

**Destabilizing force (Y_1_**)

The significance of the coefficients was tested using Student’s “t” test and the formulas are presented in Section 2.2.3. The calculated values for t are as follows: to = 457.92; t_1_ = 17.7; t_2_ = 12.83; t_3_ = 34.19; t_4_ = 40.07; t_5_ = 52.82; t_6_ = 22.29; t_7_ = 18.56. For the accuracy of the test and for adequacy, the Fischer test is performed. If t_j_ ˃ tabulated, those terms remain in the equation. The tabulated “t” is searched for the 95% (0.05) significance level and this is for Y1(force), t_tabulated_ = 2.920. Since all terms have values greater than t_tabulated_, the equation is written as follows for force:

Y_1calc_ = 79.8625 + 3.0875x_1_ − 2.2375x_2_ + 5.9625x_3_ + 6.9875x_1_x_2_ − 9.2125x_1_x_3_ − 3.8875x_2_x_3_ + 3.2375x_1_x_2_x_3_

The values for Y_1calculated_ are presented in Table 5. Whether the chosen model is good is checked with the Fisher test. F1 = 38. The tabular value of the Fisher distribution, for α = 0.05 and without any term leaving the equation Fp (f;f2), is 238.9. If F_1_ ˂ F_p_, the chosen model is good. The influence of the independent variables can be further characterized based on the values and sign of the terms

In conclusion, for the destabilization force, all terms of the equation are taken into account, so all the independent variables, x_1_, x_2_, x_3,_ influence the resistance force of the avocado and chickpea purees. The high value of the b_0_ term (Table 6) leads to the conclusion that it is considered that other compositional factors and technological parameters have an influence. Thus, the studied influence factors are the amount of chickpeas (x_1_) with a high weight because the value of the coefficient is positive and the amount of avocado (x_2_) which has less influence because the value of the coefficient is low and negative. The independent variable x_3_ has a high influence; the combination of chickpeas and avocado has a high influence. The combination of the second-order term between chickpeas and the sugar/total acidity ratio has a high influence. The variables x_1_x_2_x_3_ as a third-order factor have a high influence on the textural characteristics of the chickpea puree with avocado.

**Textural analysis from the mathematical model**

**Figure 3:**
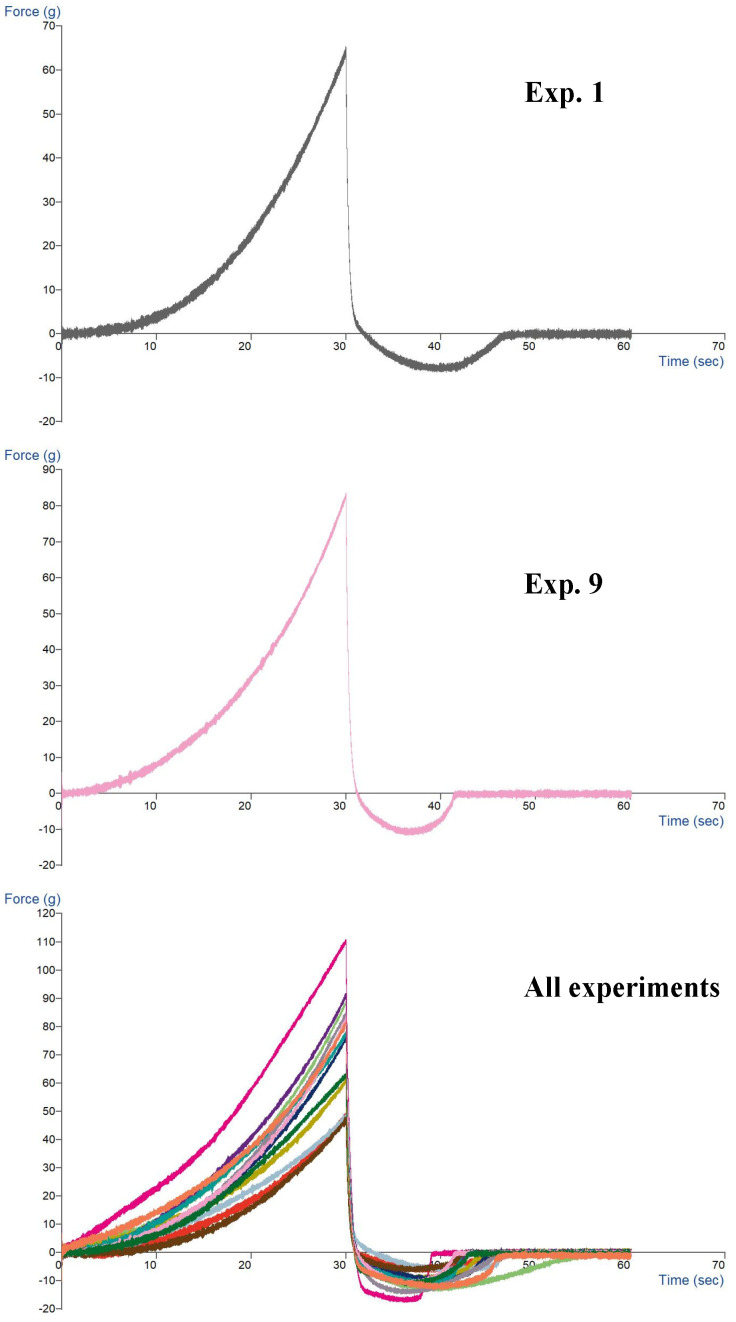
For example, textural profile of the curves of avocado–chickpea puree in the attached cone testing of the chickpea–avocado mixture and taste materials, using the TA. XT. plus C texturometer: exp 1; 9. All experiments represent the experiments created by a factorial model.

The textural properties of the variants, generated by the experimental design, were tested using the cone attached to the texturometer. The materials subjected to analysis, the purees, are semi-solid, viscous and spreadable. The graphs (Figure 3) were drawn by recording the destabilizing force (g) for each sample. The moisture of the samples is very important [40].

**Figure 2:**
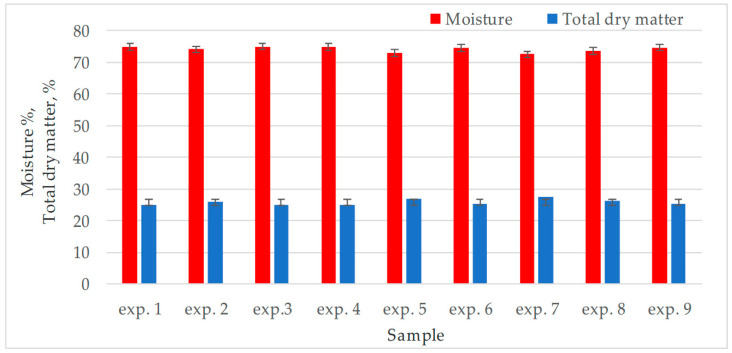
Comparative values of moisture and total dry matter of the chickpea–avocado mixture prepared according to the working variants established in the mathematical model. The values are presented as mean ± standard deviation, for all results (*n* = 3).

Consequently, the moisture (m) was analytically determined and graphically represented in Figure 2. Dry matter, which is another quality indicator, was calculated with the formula (100 m) (%) and represented in the same figure. The values for humidity are in the range 72.6–75.0%.

It has been found that the lower the moisture content and the higher the total dry matter, the higher the value of the resistance force. The textures can be varied depending on the water content. This influences the safety, the quality, and the physical properties due to the interaction with molecules such as proteins or polysaccharides. Hydrogen bonds are formed and the water molecules can be incorporated into the structure of the biopolymers. Thus, a special structure appears influences the engineering properties of the purees or sauces [41,42]. Spreadability can also be an important texture descriptor.

The textural analysis is important in controlling the quality of the puree, creating new products and correcting the recipe. The peak of firmness occurs after 30 s (end of downward run).

From the graphs (Figure 3) obtained for the different combinations of the two raw materials, avocado and chickpea, it is observed that the value of the force ranges between 59.5 g and 108.3 g, meaning that the hardness of the chickpea paste was softened by the spreadability of the avocado puree, at the same time reducing the stickiness (Table 7).

**Table 7:** Attached cone turning force values.

Experiments	* Force [g]	Distance [mm]	Time [s]
1	−7.5	25.022	34.916
2	** nD	29.835	29.842
3	−8.52	25.9525	33.899
4	−10.0	26.406	33.563
5	−16.0	2.,051	32.545
6	−9.6	25.614	34.3242
7	−11.1	25.3165	34.535
8	−14.3	27.009	32.759
9	−5.4	2.6565	34.028

*—when withdrawing the cone, the punch therefore has a negative value. **—undetermined.

The lower the hardness and firmness, the lower the stickiness, the lower the shear stress decreases and the spreadability increases, at ambient temperature (20–25 °C). In Figure 3, in the cumulative “all” plot, all graphs have a similar qualitative profile with small differences that are given by the composition of the recipes, especially by the ratio between the amount of avocado and the chickpea puree. Using the graphical results of the texturometer, the behavior of the puree was studied. Chickpea has a technological behavior that is influenced by the humidity and the presence of some contents of the recipe. Avocado has a pseudoplastic behavior, affecting the viscosity under the influence of temperature, pH and the presence of other ingredients. The mixture is considered to be an avocado-based emulsion influenced by other raw materials and ingredients or additives and can be applied in the preparation of emulsions. Chickpeas are rich in proteins and starch whereas avocados are rich in lipids. The shear rate is important, as well as the gelled structure of the chickpeas which gives water binding capacity, while the avocado gives creaminess and a lubricating effect. The chickpea/avocado ratio influences the texture.

Studies of the composition of some purees have been performed in the literature. Thus, the composition of the raw materials and their combination, chickpeas and avocado, is considered multiphasic, of oil-in-water emulsions and water-in-particles, presenting a network structure [42]. Water is a vital component that can shape the network structure, the rheology and the friction properties, the hardness, the bonding between the components, the lubricating effectiveness of the lipid droplets, the viscosity of the mixture and the texture [41].

**Sensory score (Y_2_)**

The value determined for the sensory analysis of the working variants is listed in Table 6. The values of the regression coefficients were determined using the specific formulas and are shown in Table 6. From the calculations, the values—t_0_ = 122.00; t_1_ = 2.13; t_2_ = 2.35; t_3_ = 1.64; t_4_ = 5.35; t_5_ = 1.09; t_6_ = 4.58; t_7_ = 1.21—were obtained for the significance of the coefficients in the equation for the sensory analysis. For Y_2_ (sensory score), t_tabulated_ = 2.920. All terms (t_n_) that have values lower than t_tabulated_ are canceled and the equation is written as follows for the sensory score:

Y_2calc_ = 9.9488 + 0.4363x_1_x_2_ + 0.3738x_2_x_3_

So five terms were eliminated from the original form of the equation. The values for Y_2calculated_ are presented in Table 4. When checking with Fisher’s test the mean square of the residuals, the value F = 6.58 ˂ F_tabulated_. The tabulated value of the Fisher distribution for α = 0.05 and with five terms leaving the equation F_p_ (f1;f2) is 8.85. F_2_ ˂ F_p_; thus, the chosen model is good.

The term b_0_ has an average value between the values for the sensory score which leads to the conclusion that these could be the independent variables that influence the consumer through the sensory characteristics.

The results for the sensory score were obtained using a questionnaire for panelists based on the standard method of sensory analysis presented in detail in the materials and methods chapter.

In the study of the sensory note, according to the mathematical model, some terms of the equation were eliminated. The strong and positive influencing factors remained the second-order element, the combination between the independent variables x_1_ (the amount of chickpeas), x_2_ (the amount of avocado), but equally important is the combination of the amount of avocado (x_2_) and the ratio between the amount of sugar and acids (x_3_).

**Figure 4:**
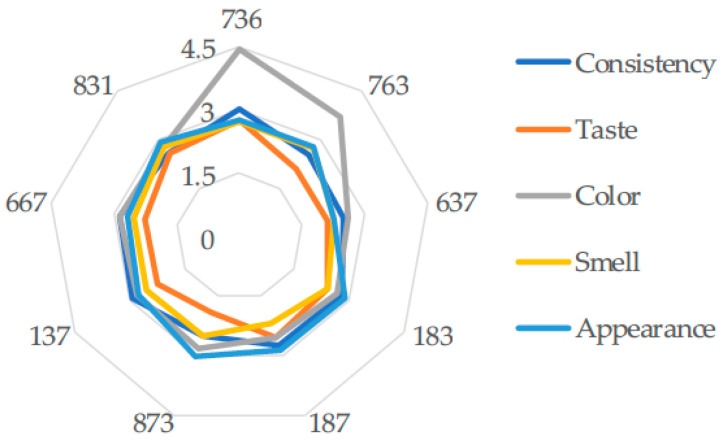
Comparative graphic representation of the values of the sensory characteristics (arithmetic mean) of the studied variants according to the chosen mathematical model: 736—exp.1; 763—exp.2; 637—exp.3; 183—exp.4; 187—exp.5; 873—exp.6; 137—exp.7; 667—exp.8; 831—exp.9, 9′, 9′′.

According to the panelists’ response, the values obtained for consistency (textural characteristic), as a score, are between 2.49 out of 5 and 3.03 out of 5 points, very close to the studied experiments (*p* ˂ 0.05). The color characteristic, according to the graph in Figure 4, has a special value for the working variants 736 (4.43 out of 5 points) and 763 (3.71 out of 5 points). The values for taste are between 1.92 out of 5 and 2.78 out of 5 points.

The variant with the sensory score of 11 points, i.e., obtained after conducting experiment 1, is recommended. Consistency had the highest value for variant 736 (3.03p out of 5p) (exp.1). The evolution of the sensory score in ascending order is as follows:

637 (exp.3) ˂ 873(exp.6) ˂ 183 (exp.4) ˂ 763(exp.2) ˂137(exp.7) ˂ 831 (exp.9) ˂ 667 (exp.8) ˂ 187 (exp.5) ˂ 736 (exp.1).

This ratio was modeled using the factor analysis to optimize the production recipe. The model also refers to the impact on the consumer by determining the sensory note and on the sugars/total acidity ratio as an influence of the other components of the recipe on the recipe, but with an alteration in the organoleptic characteristics.

The Pearson correlation coefficients between force (instrumental) and sensory descriptors of the texture, calculated for a significance level of α = 0.05, are shown in Table 8. The results of the correlations for all combinations given by the sensory characteristics of the purees is a significant large negative relationship between the destabilizing force and the sensory perceptions. *p*-value generally has very small values, i.e., *p* ˂ α (0.05) so the hypothesis from which it is started is supported. T-test statistic has negative values which means that the values are not in the acceptance zone. In this case, it can be said that with the decrease in the value of the destabilizing force, the consumer’s behavior changes. For more concrete results, it would be better to use at least double the number of determinations, although, in this case, the results were also significantly variable (*p* ˂ 0.05). Other studies have shown the correlation between instrumental and sensory values of textural descriptors. Thus, with the increase in the value of the destabilization force, the sensory score decreases. This is because the effort made by the panelist to chew for swallowing is greater, more uncomfortable, for example, with carrot puree [43]. In other studies, the presence of the avocado pulp in a sauce did not greatly influence the values of the sensory note, overall, for the characteristics assessed regardless of the used analysis method, either analytical or preferential, the differences being in color and aspect [7].

**Total acidity** (Y_3_)

The values determined for the total acidity of the working variants are given in Table 5. The values of the regression coefficients were determined using the specific formulas specified above and are given in Table 5. From the calculations, the following values were obtained for the significance of the coefficients in the equation for total acidity: t_0_ = 288.56; t_1_ = 25.36; t_2_ = 17.01; t_3_ = 13.80; t_4_ = 20.22; t_5_ = 43.33; t_6_ = 19.58; t_7_ = 28.57. For Y_3_ (total acidity), t_tabulated_ = 2.920. All terms (t_n_) that have values lower than t_tabulated_ disappear from the system. However, here, all terms have values higher than t_tabulated_; the equation is written as follows for total acidity.

Y_3calc_ = 1.1238 + 0.0988x_1_ − 0.0663x_2_ + 0.0538x_3_ − 0.0788x_1_x_2_ + 0.1688x_1_x_3_ + 0.0763x_2_x_3_ + 0.1113 x_1_x_2_x_3_

The values for Y_3calculated_ are presented in Table 5. When checking with the *Fisher* test, the mean square of the residuals s^2^_r_ = 0.1355, and the value F_3_ = 1.353 ˂ F_tabulated_. The tabulated value of the *Fisher* distribution, for α = 0.05 and without any term leaving the equation F_p_ (f_1_; f_2_), is 238.1. Since F_3_ ˂ F_p_, the chosen model is good. In conclusion, for the total acidity, all the terms of the equation are taken into account, including the independent factors such as avocado, chickpeas, acid, sugar and their combination as elements of the recipe. The b_0_ term has an average value (1.1238) that is, between the values for total acidity, which confirms that these raw materials contribute to the value of the sugars/acid ratio with the increase in the numerator for the increase in the total acidity value.

Total acidity is an important indicator also involved in the creation of the taste descriptors. The raw and auxiliary materials in the recipe contribute to the number of acid groups determined by neutralization with a base that has a given concentration. According to the regression equation, the value of total acidity is greatly influenced (the positive value of the coefficient of the term x_1_) by the amount of chickpeas, the ratio between sugars and vinegar (“+x_3_”), the combination of chickpeas and vinegar in the recipe (“+x_1_x_3_”), avocado, sugars and vinegar (“+x_2_x_3_”) as well as the third-order factor, i.e., the combination of components such as chickpeas, avocado, sugar and vinegar with a constant addition of salt and garlic. With a negative sign, less influential terms were the amount of avocado (“−x_2_”) and the second-order factor, the amount of chickpeas and avocado (“−x_1_x_2_”). Total acidity is within the range of 0.8–1.620 g/100 g expressed in acetic acid. In another study of the preparation of a tomato sauce with avocado, total acidity had values between 1.02 and 1.24 g/100 g, expressed in acetic acid, and was lower than the amount of avocado [7].

The protein–lipid interaction is explained by the creation of a weak network in the presence of the fats in the avocado. The interactions are influenced by the presence of the ions (from the acidic substances in the recipe) and the pH. According to the sensory analysis (Figure 4) for the balanced taste, close values were obtained. Experiment 6 has the lowest acidity value, the ratio between the amount of sugar and the amount of added acetic acid is 42.5. Experiment 1, which received the best sensory vote, had an acidity of 0.960 g/100 g, so with a sugar/acidity ratio of 32.5, meaning an amount of 2.6% sugar and 6.9% vinegar.

## 4. Discussion

The fluidity and the flow of the obtained puree do not exist. It does not flow. The proportion of avocado and chickpea pulp is variable. The force value [g] has shifted toward an increase to 108.3 g or a decrease to 63.7 g. The value of the destabilizing force in other studies on the textural quality of carrot puree with different thickening agents is between 107.88 g and 118.62 g [27]. This phenomenon shows that the structural network formed with an addition of chickpea leads to changes in hardness. The raw materials that were used are finely ground. This mechanical operation contributes to the extracellular release of components and these contribute to the formation of the specific structure and influence the consolidation or the destabilization of the puree. This aspect is also influenced by the independent variables according to the chosen model (recipe), by the operational parameters, exactly what was sought. These aspects have also been encountered in other studies on purees and the influence of avocado pulp on the mechanical properties of the purees was found [44,45]. These texture parameters are considered very important when assessing the force required for the first bite (mastication), when consuming this puree and for swallowing. The higher the percentage of avocado, the higher the number of soft particles, the lower the viscosity. These effects in the current study and other studies [27] are valid for a soft viscoelastic solid structure. The strain is represented by the adhesion at the contact points [27] and when the cone is removed, the negative value is recorded (Table 6), which means stickiness is rather low since the values range between −5.4 g and −16.0 g. There were no significant differences between the tested samples. For the sensory analysis, the value of the sugar/acidity ratio influences the sensitization of the salivary glands and the swallowing of the food bolus, even if the individual differences are not significant [27].

## 5. Conclusions

The goal was achieved by identifying the recipe components for which the network structure, textural properties and physicochemical indicators are the best. The recipe with a chickpea puree/avocado puree content ratio of 1:1 and a sugar/acetic acid ratio of 32.5 corresponding to the experiment 1 is the best variant, which was preferred by consumers. The suggested influencing factors are relevant and this aspect was clearly highlighted by the mathematical modeling. For the sensory note, from the remaining terms, only those of the second-order influenced, namely the combination between chickpea and avocado and, on the other hand, that between avocado and sugar/acetic acid. The appearance characteristic was noted with the descriptors: creaminess, spreadability and color. For the total acidity, the most important influencing factor, positive and with the highest value, was the combination between the amount of chickpea and the sugar/acetic acid. The maximum value is 1.2 g/100 g expressed as acetic acid. The most important factors influencing the destabilizing force are the sugar/acetic acid ratio, the amount of chickpeas and avocado, and the third-order factor, i.e., the combination of the three. The maximum recommended value for the destabilizing force is between 60 and 80 g. It is recommended to use the recipe—chickpea puree/avocado puree (1:1), sugar 1.6%, salt 1.6%, garlic 1.4%, vinegar 0.8%—for mass production, taking into account the influencing factors.

The finely ground raw materials and spices led to the release of cellular components that influenced the formation of special sensory and textural characteristics. For mass production, it is recommended to use a technological line for the production of mustard or ketchup.

## Figures and Tables

**Table 1:** Basis for assessing organoleptic characteristics.

Rating	Basis for Assessing Organoleptic Characteristics	Number of Points
Very good	Specific positive characteristic, very well-defined, without defects	5
Good	Specific positive characteristic, fairly well-defined, with very small defects	4
Satisfactory	Specific positive characteristic, very poorly defined, with small defects due to which it is at the minimum level allowed by the standard	3
Unsatisfactory	Presents deficiencies or defects of the characteristic due to which it does not meet the minimum condition of the standard; the product can be used for direct consumption	2
Inadequate	Presents defects, obvious deficiencies of the characteristic that it can no longer be used for consumption except after appropriate processing	1
Altered	Presents accentuated defects of the characteristic, specific to an altered product that can no longer be consumed	0

**Table 2:** Quality indicators determined for canned chickpeas in brine and fresh avocado.

Quality Indicators	Canned Chickpeas	Avocado Fruit
Net mass, g	425 ± * 21.79	247.3 ± 5.1
Liquid fraction, %	42.6 ± 2.02	-
Solid fraction, %	57 ± 2.00	-
Circumference, cm	-	27.9 ± 9.13
Fruit length, cm	-	12.7 ± 2.494
Peel, %	-	15.16 ± 0.78
Stone, %	-	14.79 ± 0.406
Pulp, %	-	69.24 ± 0.360
pH	4.58 ± 0.01	6.34 ± 0.09
Titratable acidity, g/100 g expressed as citric acid	0.20 ± 0.03	0.12 ± 0.034
Total soluble solid, ^o^Bx	9.60 ± 0.23	-
Vitamin C, mg/100 g	2.26 ± 0.41	15.1 ± 1.35
Salt, %	0.29 ± 0.016	-
Moisture, %	66.53 ± 0.83	88.66 ± 0.38
Total dry matter, %	33.47 ± 0.81	10.9 ± 0.33

* Values are presented as mean, ± standard deviation for all results (*n* = 3).

**Table 3:** Variable level code for 100 g of puree.

		Variable Level Code		
Variable	Symbol	−1	0	+1
Chickpea content	x_1_	45	50	55
Avocado content	x_2_	45	50	55
Sugar/vinegar ratio	x_3_	32.5	37.5	42.5

**Table 4:** Quantities (g) (independent variables) for the practical implementation of the experiments from the chosen mathematical model.

Experiments and Macroscopic Images		x_1_	x_2_	x_3_
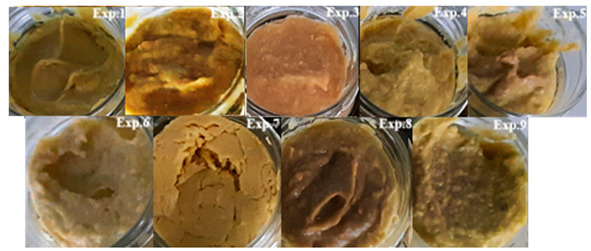	1	450	450	32.5
	2	550	450	32.5
	3	450	550	32.5
	4	550	550	32.5
	5	450	450	42.5
	6	550	450	42.5
	7	450	550	42.5
	8	550	550	42.5
	9	500	500	37.5
	9′	500	500	37.5
	9′′	500	500	37.5

**Table 8:** Correlation between force (instrumental) and sensory descriptors of the texture of chickpea and avocado purees.

Texture Attribute	Sensory Attribute	Pearson Correlation Coefficient (r)	R^2^	*p*-Value	T Statistic	Distribution	Significance Level (α)
Force(g)	Consistency(points)	−0.8239	0.6788	0.00002636	−5.8145	Two-tailed	0.05
Force(g)	Appearance(points)	−0.8072	0.6516	0.0000514	−5.4698	Two-tailed	0.05
Force(g)	Taste(points)	−0.6912	0.4777	0.001491	−3.8254	Two-tailed	0.05
Force(g)	Color(points)	−0.9281	0.8613	2.872 × 10^−8^	−9.968	Two-tailed	0.05
Force(g)	Smell(points)	−0.8072	0.6516	0.0000514	−5.4698	Two-tailed	0.05

## Data Availability

The original contributions presented in this study are included in the article and supplementary material. Further inquiries can be directed to the corresponding author.

## Supplementary Materials

The following supporting information can be downloaded at: https://www.mdpi.com/article/10.3390/foods14234082/s1.
