# Supplementary material for: Applying Factorial Modeling to the Optimization of Textural Descriptors and Sensations Relative to Obtaining the Experimental Prototype of Chickpea Puree with Avocado
Source: Foods. 2025 Nov 28;14(23):4082. doi: 10.3390/foods14234082 (PMC12692110; doi:10.3390/foods14234082)
Supplement: Supplementary file 1 [file foods-14-04082-s001.zip › foods-3976378-supplementary.pdf]

Table A Questionnaire to fill out for the analysis of chickpea puree with avocado

Sample code number	Organoleptic characteristics scoring range, number of points awarded				
	Consistency0...5	Taste 0...5	Color 0...5	Smell 0...5	Appearance 0...5
736					
763					
637					
183					
187					
873					
137					
667					
831					

Tabel B. Basis for assessing organoleptic characteristics

Calificativul	Basis for assessing organoleptic characteristics	Number of point
Very good	Specific characteristic positive, very well defined, without defects	5
Good	Positive specific characteristic, fairly well-defined, with very small defects	4
Satisfactory	Positive specific characteristic, very poorly defined, with small defects due to which it is at the minimum level allowed by the standard	3
Unsatisfactory	Presents deficiencies or defects of the characteristic due to which it does not meet the minimum condition of the standard, the product can be used for directed consumption	2
Inadequate	Presents defects, obvious deficiencies of the characteristic so that it can no longer be used for consumption except after appropriate processing	1
Altered	Presents accentuated defects of the characteristic, specific to an altered product that can no longer be consumed	0
